# Use of a life-size three-dimensional-printed spine model for pedicle screw instrumentation training

**DOI:** 10.1186/s13018-018-0788-z

**Published:** 2018-04-16

**Authors:** Hyun Jin Park, Chenyu Wang, Kyung Ho Choi, Hyong Nyun Kim

**Affiliations:** Department of Orthopaedic Surgery, Kangnam Sacred Heart Hospital, Hallym University College of Medicine, 948-1, Dalim-1dong, Youngdeungpo-gu, Seoul, 150-950 South Korea

**Keywords:** 3D-printed spine model, Pedicle screw instrumentation, Beginners, Training

## Abstract

**Background:**

Training beginners of the pedicle screw instrumentation technique in the operating room is limited because of issues related to patient safety and surgical efficiency. Three-dimensional (3D) printing enables training or simulation surgery on a real-size replica of deformed spine, which is difficult to perform in the usual cadaver or surrogate plastic models. The purpose of this study was to evaluate the educational effect of using a real-size 3D-printed spine model for training beginners of the free-hand pedicle screw instrumentation technique. We asked whether the use of a 3D spine model can improve (1) screw instrumentation accuracy and (2) length of procedure.

**Methods:**

Twenty life-size 3D-printed lumbar spine models were made from 10 volunteers (two models for each volunteer). Two novice surgeons who had no experience of free-hand pedicle screw instrumentation technique were instructed by an experienced surgeon, and each surgeon inserted 10 pedicle screws for each lumbar spine model. Computed tomography scans of the spine models were obtained to evaluate screw instrumentation accuracy. The length of time in completing the procedure was recorded. The results of the latter 10 spine models were compared with those of the former 10 models to evaluate learning effect.

**Results:**

A total of 37/200 screws (18.5%) perforated the pedicle cortex with a mean of 1.7 mm (range, 1.2–3.3 mm). However, the latter half of the models had significantly less violation than the former half (10/100 vs. 27/100, *p* < 0.001). The mean length of time to complete 10 pedicle screw instrumentations in a spine model was 42.8 ± 5.3 min for the former 10 spine models and 35.6 ± 2.9 min for the latter 10 spine models. The latter 10 spine models had significantly less time than the former 10 models (*p* < 0.001).

**Conclusion:**

A life-size 3D-printed spine model can be an excellent tool for training beginners of the free-hand pedicle screw instrumentation.

## Background

Pedicle screws are frequently used in spine surgeries, and their use is expected to increase as the number of spinal fusion surgeries is rapidly increasing [1–4]. Pedicle screw fixation is beneficial in achieving mechanical stabilization during bony fusion. However, inadvertent perforation of pedicle screws into the spinal canal can sometimes be fatal [5]. It can lead to neurologic injury or unsatisfactory degrees of stabilization [6, 7]. The rate of pedicle screw malpositioning ranges from 0 to 25%, depending on the case’s degree of complexity and the surgeon’s level of experience [8–12]. Safe and accurate instrumentation of pedicle screws is important, and this technique is one of the major skills that a trainee in spine surgery has to learn and acquire.

Training beginners of this technique through surgical procedures of patients in the operating room is limited because of issues related to patient safety and surgical efficiency [13]. Training on cadaver spines can be an appropriate alternative, but due to high costs and lack of available cadavers for all trainees, trainers seek for other surrogate spine models [14–16]. We believe that a life-size 3D-printed model can be an excellent solution. Actual osseous spine anatomy can be reproduced into a life-size 3D-printed model allowing surgeons a firsthand look at what they will be operating on before the real surgery. This allows simulation surgery before the real surgery, increasing the learner’s ability to retain surgical skills and building learner confidence in a low-stress environment [17, 18]. Furthermore, 3D printing enables training on a life-size replica of deformed spine or young-aged spine, which is difficult to perform in the usual cadaver or surrogate plastic models.

3D printing technologies are common in product design industries, and their use is increasing in all fields [19, 20]. Recent technical developments and their popularity within the general public are leading the world to an era of personalized 3D printing, similar to what has become of a personalized computer or printer. As the popularity of 3D printing is increasing, it is becoming financially feasible and accessible to use the practice of medicine [20]. As this technology enables replication of actual osseous anatomy, it can be most beneficial to surgeons who operate on bony structures, including the spine [21]. We believe this technology can be useful in educating residents of their surgical skills.

To our knowledge, no reports have described surgical skill training of the pedicle screw instrumentation technique using a life-size 3D-printed spine model. The purpose of this study was to evaluate the educational effect of using a life-size 3D-printed spine model for training beginners of the free-hand pedicle screw instrumentation technique in improving screw instrumentation accuracy and procedure time.

## Methods

### 3D printing of life-size spinal models

#### CT scan

**Fig. 1:**
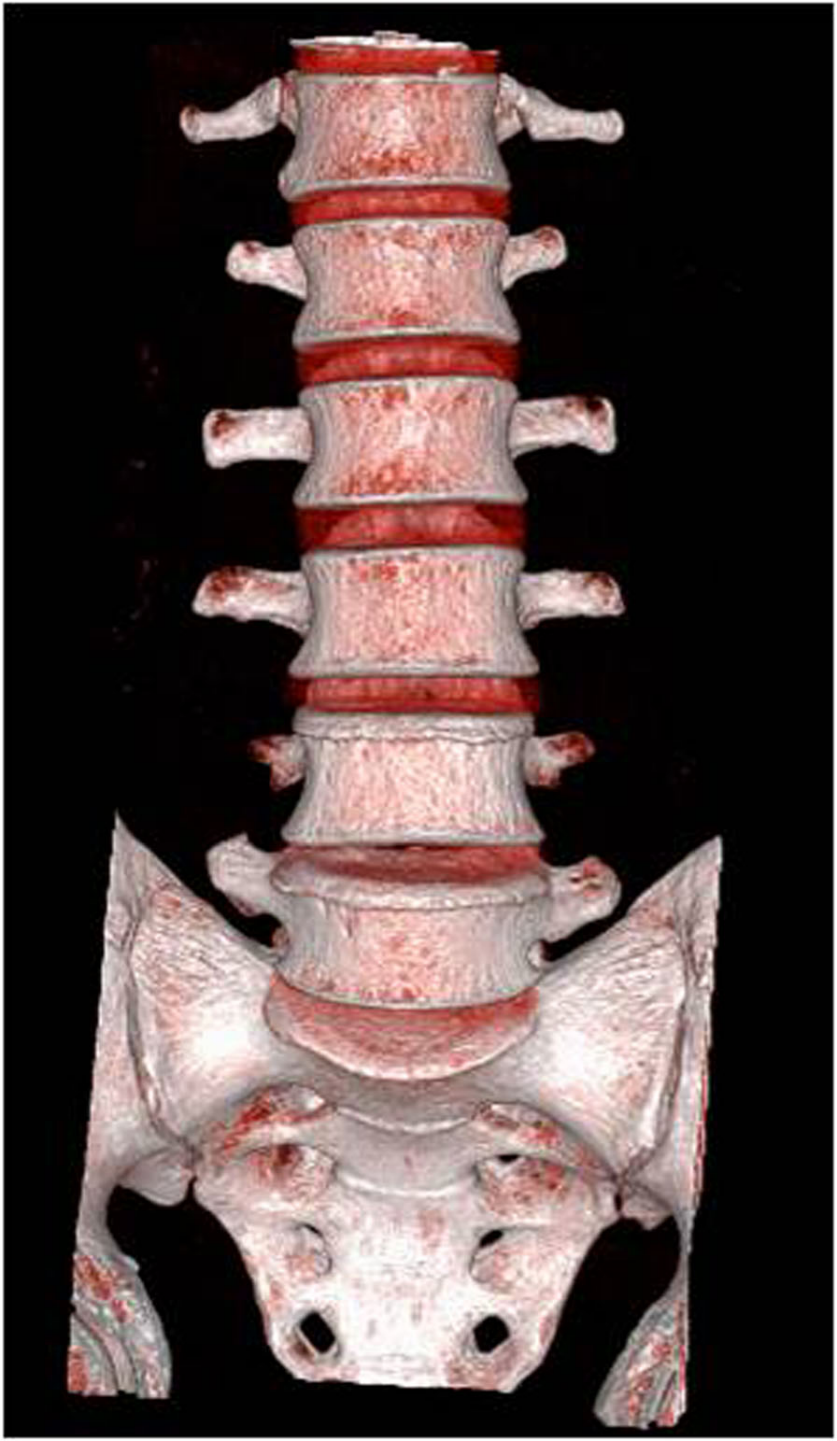
A CT scan was taken from the lower endplate of 12th thoracic vertebra (T12) and below the 1st sacral vertebra (S1) with a 1-mm thickness slice

Ten adult patients (5 male, 5 female) with low back pain were enrolled. The mean age of patients was 35.2 years (range, 24–52 years). They were confirmed not to have any congenital abnormalities, other deformities, and instability on the lumbosacral spine by using plain radiographs and CT scan. Patients underwent CT scan from the lower endplate of the 12th thoracic vertebra (T12) and below the 1st sacral vertebra (S1) with a 1-mm thickness slice (Fig. 1). Institutional review board approval was obtained for the study.

#### 3D printing

**Fig. 2:**
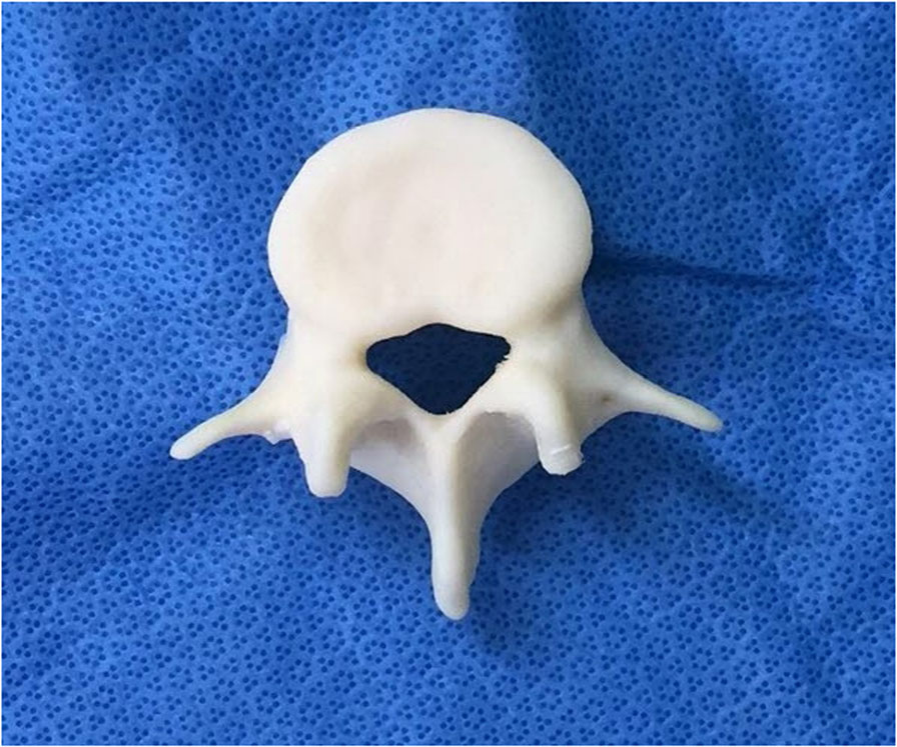
A real-sized lumbar spinal model was produced using the 3D printing machine (Objet30Pro®, Stratasys, Valencia, CA, USA). Five lumbar models were produced for one spine model. In total, 20 spine models (100 lumbar models) were produced

The data acquired from the CT scan were stored in the Digital Imaging and Communications in Medicine format and converted to a standard triangulation language file format by using a specialized software (MIMICS: Materialise Interactive Medical Image Control System Software, Materialise, Leuven, Belgium) that can be used by the 3D printing machine (Objet30Pro®, Stratasys, Valencia, CA, USA) to produce a life-size lumbar spine model with polypropylene (Fig. 2). Twenty life-size 3D-printed lumbar spine models were made for the study.

### Pedicle screw fixation in life-size 3D-printed lumbar spine models

**Fig. 3:**
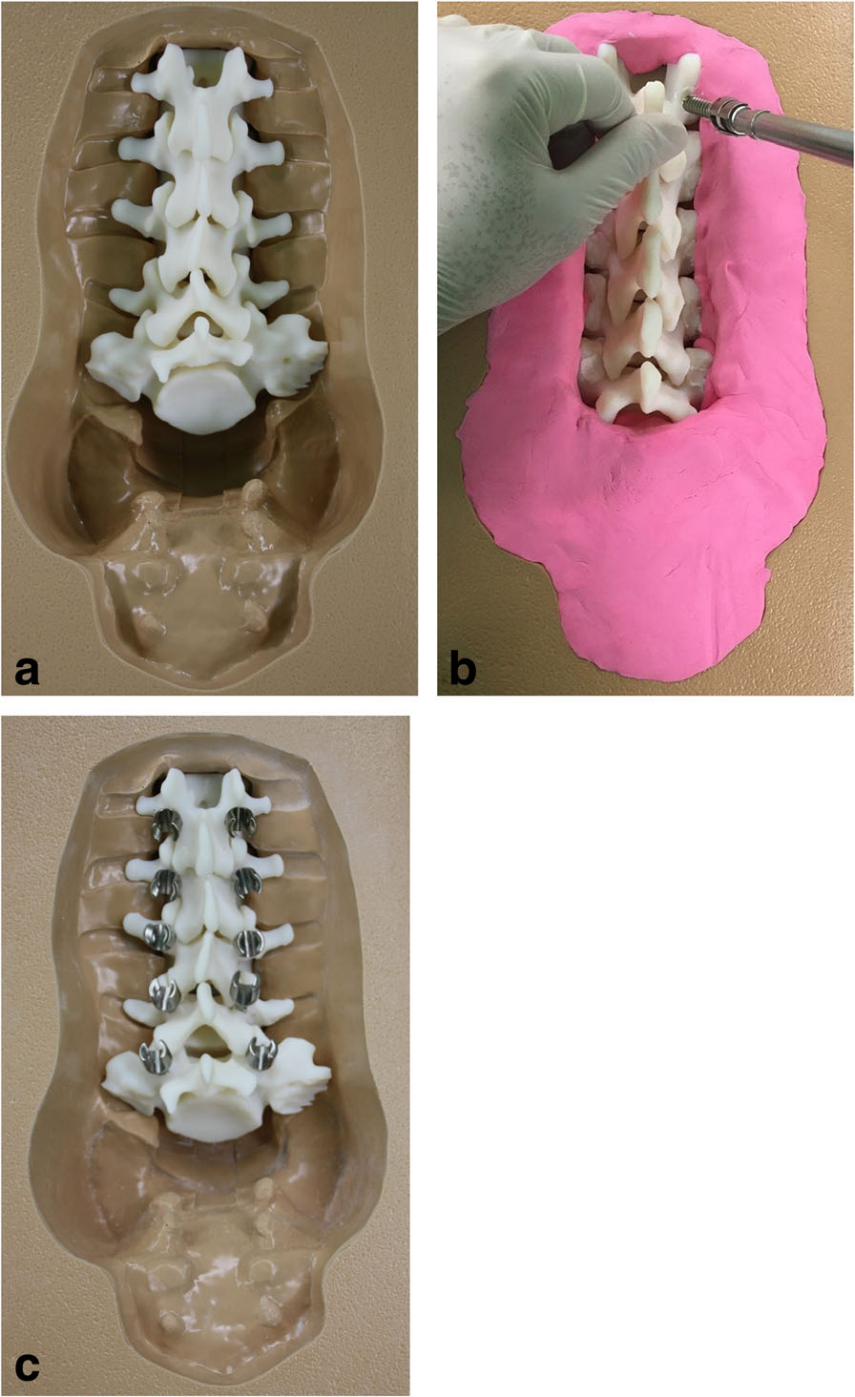
**a** Each 3D-printed spine model was mounted on the lumbar spine holder (Sawbones®, Vashon Island, WA, USA) to secure each vertebral body. **b** Synthetic polymer clay was placed surrounding the pedicle; thus, only the posterior surface anatomy could be seen, but the pedicle size or orientation could not be seen. **c** Two novice surgeons who had no experience of free-hand pedicle screw insertion technique were instructed of the technique by an experienced surgeon, and each inserted 10 pedicle screws for each lumbar spinal model

**Fig. 4:**
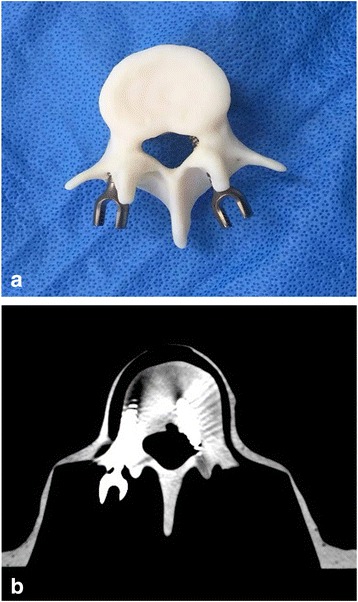
**a** The spinal models underwent **b** CT scan immediately after the pedicle screws were inserted to evaluate their instrumentation accuracy

Two residents who had no experience of pedicle screw instrumentation were selected to participate in this study. First, the residents were instructed by an experienced spine surgeon (KJC) on the instrumentation technique of pedicle localization and the method of pedicle screw fixation. Subsequently, each 3D-printed spine model was mounted on the lumbar spine holder (Sawbones®, Vashon Island, WA, USA) to secure each vertebral body with the L3 vertebra placed most ventrally (Fig. 3a). Synthetic polymer clay was placed surrounding the pedicle; thus, only the posterior surface anatomy could be seen, but the pedicle size or orientation could not be seen (Fig. 3b). Two novice orthopedic residents were provided with plain radiographs and CT scan images on each patient’s lumbar spine, then each inserted 10 pedicle screws (two screws for one vertebra) for one spine model (5 lumbar vertebrae). The residents first inserted a K-wire on the entry point of the pedicle screw, which was at the junction of the midline of the transverse process and the lateral margin of the facet joint. A small pilot hole was made with an awl. After determining the ideal pathway for the screw by using a guide wire, a hole was made with a small-diameter drill. The opening for the entrance of the pedicle screw was checked with a small ball tip probe. The safety of the pathway for the pedicle screw was determined when an intraosseous resistance was noted in all (medial, lateral, superior, and inferior) directions. Drilling was further carried out using tappers with larger diameters up to 5 mm. Finally, the pedicle screw was gently inserted (Fig. 3b). Each resident inserted pedicle screws into 10 3D-printed spine models (100 pedicle screws for one resident) (Fig. 3c). The residents examined each specimen after instrumentation to identify their errors to improve their accuracy on the next one (Fig. 4a).

### Radiologic analysis of pedicle screw instrumentation

The spinal models underwent CT scan immediately after the pedicle screws were inserted to evaluate their instrumentation accuracy (Fig. 4b). Screw malposition and breach of medial and lateral wall of the pedicles were recorded. Position of the screws were classified into one of four categories based on their position relative to the pedicle: category A, fully contained within the pedicle; category B, breach less than 2 mm; category C, breach of 2 to 4 mm; and category D, breach greater than 4 mm. A critical violation was defined as > 2 mm. Perforation of the pedicle wall > 2 mm is reported to increase the potential for neurologic complications. These results were interpreted and recorded by expert musculoskeletal radiologists blinded to this study. In addition, the length of time to complete the procedure was recorded. The results of the latter 10 spinal models were compared with those of the former 10 models to evaluate learning effect. The resident, who has done the screw instrumentation just after their procedure and before the new specimen, was informed of the pedicle screw instrumentation result on each spine model (10 pedicle screws inserted in 5 vertebrae); hence, their experience can help with the next procedure.

### Statistical analysis

Statistical analysis was performed using SPSS version 23.0 (SPSS, SPSS Inc., Chicago, IL, USA) software. Data normality was assessed by the Kolmogorov–Smirnov test. Chi-square test was used to compare the incidence of screw violation of the pedicle between the former 10 (100 pedicles) and latter 10 spine models (100 pedicles). Mann–Whitney *U* test was used to compare the length of time to complete the screw instrumentation between the former and latter 10 spine models. Logistic regression analysis was performed to evaluate improvement in error rates through the 20 spinal models with respect to the total error rates. Bivariate associations between the vertebral level and the pedicle screw violation were examined by using Spearman’s correlation analysis. Statistical significance was accepted for *p* values < 0.05.

## Results

### Pedicle screw violations

Two-hundred pedicles in 100 vertebral bodies were inserted with 5-mm cylindrical pedicle screws. A total of 37/200 screws (18.5%) perforated the outer cortex of the pedicles with a mean of 1.7-mm violation (range, 1.2–3.3 mm). Of the 37 perforating screws, 36 (97%) violated the medial side of the pedicle. The first and second residents made 18 (49%) and 19 (51%) violations, respectively, and no significant difference was found between the two (*p* > 0.05).

No screw was classified in category D (> 4-mm cortical breach). However, blinded CT evaluations of screw placement indicated that 5.5% (11/200), 13% (26/200), and 81.5% (163/200) of screws were in categories C (2- to 4-mm breach), B, and A, respectively. When 20 spine models were divided into two groups (the former and latter 10 spine models), the former 10 spine models had 11% (11/100), 16% (16/100), and 73% (73/100) of screws in categories C, B, and A, respectively. The latter 10 spine models had 0% (0/100), 10% (10/100), and 90% (90/100) of screws in categories C, B, and A, respectively (Table 1).

**Table 1:** Screw placement accuracy

Screw placement category	Percentage of screws	
	Former group (*n* = 100 screws)	Latter group (*n* = 100 screws)
A (fully contained)	73	90
B (breach < 2 mm)	16	10
C (breach of 2–4 mm)	11	0
D (breach > 2 mm)	0	0

Former group: 10 3D-printed spinal models that residents instrumented pedicle screws earlier to the 10 latter group. Latter group: 10 3D-printed spinal models that residents instrumented pedicle screws later to the 10 former group

Less percent total violations were seen in the latter 10 spine models (10/100 pedicle screws) compared with the former 10 spine models (27/100 pedicle screws) (*p* < 0.05; odds ratio, 0.30; 95% CI, 0.137–0.661) (Fig. 1). No critical violation of the pedicle screws (> 2 mm) were seen in the latter 10 spine models (0/100 pedicle screws) compared with the former 10 spine models (11/100 pedicle screws).

### Incidence and degree of violation based on vertebral level

Violations occurred in all levels of the lumbar spine except at L5 (Table 2). The most common level of violation occurred at L1 with 32.5% (13/40). L2, L3, and L4 had 25% (10/40), 22.5% (9/40), and 12.5% (5/40), respectively, with L4 having the least number of violations (Table 2). Pedicles in the lower vertebral level had less percent violation compared with higher vertebra level (Spearman’s correlation coefficient, − 0.28; *p* < 0.01). Based on the lumbar spine level, violations above and below L3 were 86% (32/37) and 14% (5/37), respectively, and a significant difference was found (*p* < 0.05).

**Table 2:** Results of 20 spinal models with 200 lumbar pedicles instrumented

	No. pedicles instrumented	No. violations	% violation	Avg. violation (mm)	Range of violation (mm)	No. critical violation	% critical violation
L1	40	13	32.5	1.85	1.3–2.5	5	12.5
L2	40	10	25	1.87	1.2–3.1	4	10
L3	40	9	22.5	1.74	1.2–3.3	2	5
L4	40	5	12.5	1.44	1.3–1.8	0	0
L5	40	0	0	0	0	0	0

Critical violation (> 2 mm) did not occur in L4 and L5. The most common level of critical violation occurred at L1 with 12.5% (5/40). L2 and L3 had 10% (4/40) and 5% (2/40) violations, respectively. Pedicles in the lower vertebral level had less percent violation compared with higher vertebral level (Spearman’s correlation coefficient, − 0.22; *p* < 0.01). Violations more than 4 mm of the pedicles did not occur.

### Length of procedure for pedicle screw placement

**Fig. 5:**
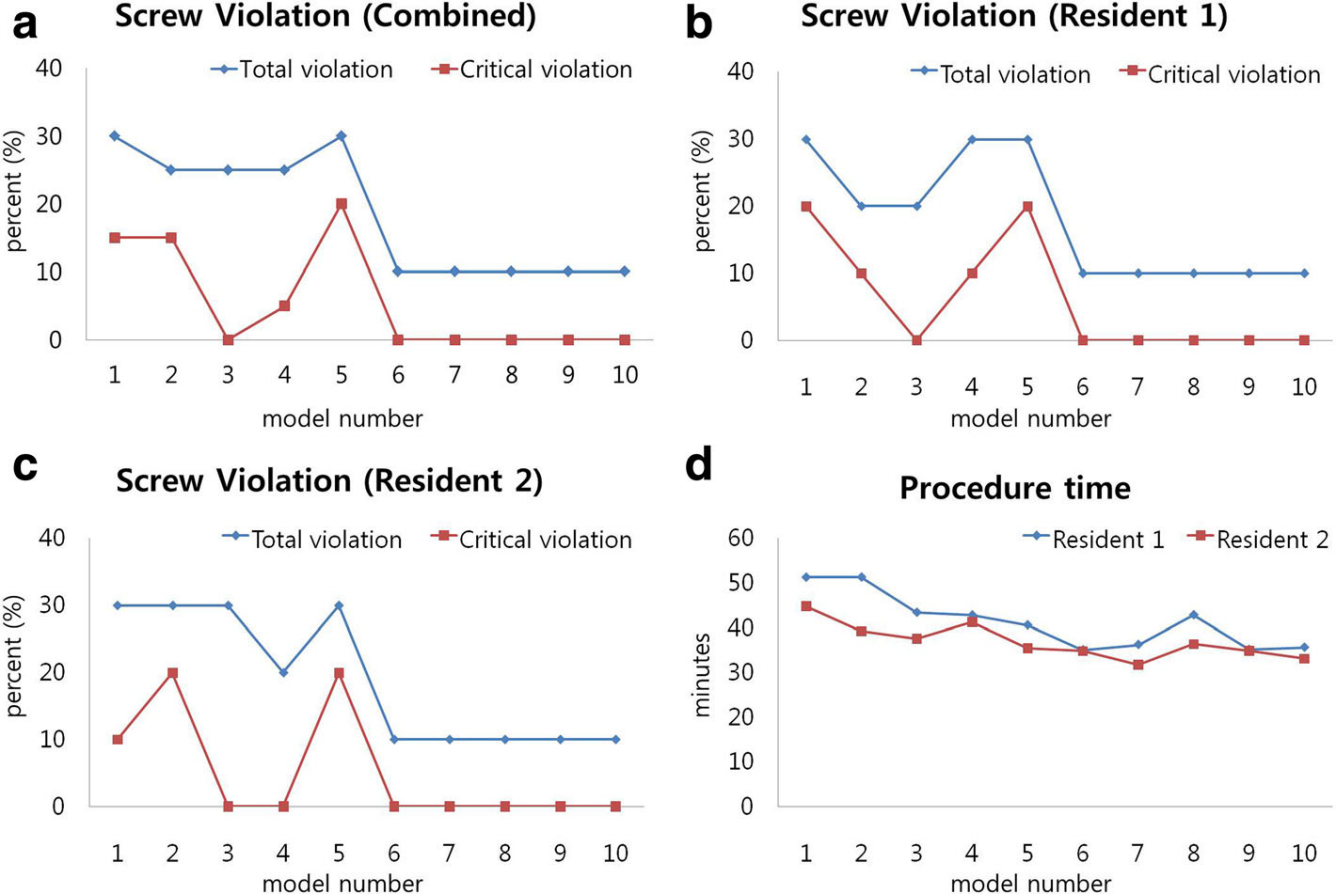
Total pedicle violation percentage, critical (breach of >2mm) pedicle violation percentage are shown for resident 1 (**b**), resident 2 (**c**), and combined (**a**). (**a**) The mean total violation percentage decreased from 30% with the first spine model to 10% after completing five spine models (50 pedicle screws). This violation percentage became stable at 10% from the sixth to tenth models. The mean critical violation (>2-mm breach) percentage also decreased as residents continued practicing and became stable at 0% after completing five models. (**d**) The length of time required to complete screw instrumentation decreased as residents continued to practice the skills on the 3D-printed models. A strong negative correlation was observed between the repetitive time of screw instrumentation and length of procedure (Spearman’s correlation coefficient, −0.71; *p* < 0.001)

The mean length of time to complete 10 pedicle screw instrumentations in a spine model was 42.8 ± 5.3 min for the former 10 spine models and 35.6 ± 2.9 min for the latter 10 spine models (Table 3). The latter 10 spine models required significantly less time than the former 10 spine models (*p* < 0.001). Later instrumentation of the pedicle screws required less time compared with earlier instrumentation (Spearman’s correlation coefficient, − 0.71; *p* < 0.001) (Fig. 5).

**Table 3:** Procedure time required for fixation of 10 pedicle screws for each model

No.	Resident 1	Resident 2
1	51.19	44.48
2	51.21	39.13
3	43.26	37.36
4	42.50	41.14
5	40.38	35.28
6	35.06	34.49
7	36.18	31.44
8	42.53	36.21
9	35.11	34.52
10	35.41	33.06

Numbers presented as minutes

*No.* numbers of the real-sized 3D-printed spinal models

## Discussion

Pedicle screw is a penetrating type of fixation device and offers a secure vertebral grip that enhances control of the inserted segments and firm fixation [11, 22]. After it has gained popularity, the number of spinal fusion has exceedingly increased for the last few decades. Spinal fusion using pedicle screw fixation became a gold standard technique, but pedicle screw instrumentation has its own risk. Complications such as neurological injury, spinal construct failure, and deep wound infection have been reported by researchers [6, 7]. Moreover, great vessel injury caused by malposition of the pedicle screw may cause fatal results in the thoracic spine in patients undergoing deformity correction [5] Safe and efficient technique for pedicle screw instrumentation is essential [23, 24].

Many techniques have been reported during several decades [22, 25, 26]. Parker et al. reported the accuracy and safety of free-hand technique for pedicle screw instrumentation in thoracic and lumbar spine [10]. Currently, more accurate pedicle screw instrumentation is possible with the aid of computer-assisted navigation systems [8, 9, 12, 26]. Although advanced scientific devices are beneficial, having surgeons well accustomed to anatomy and the applied technique is essential [27, 28]. This is more important to residents who are not familiar with surgical skills and when mistakes can cause fatal results, such that can occur during pedicle screw instrumentation [20, 21].

However, learning the technique is technically demanding even for clinical fellow surgeons. For this reason, Bergeson et al. insisted that surgeons who do not have enough experience in pedicle screw instrumentation should practice the technique with cadavers before the real surgery [23]. Training can be safely performed in a low-stress environment using cadavers and synthetic bone. However, getting enough cadavers for teaching and training of pedicle screw instrumentation is difficult. Even when surgical simulation training on cadavers or synthetic bone is over, the opportunity to visualize and handle a replica of the spine before surgery can be enormously helpful in building confidence. A life-size 3D-printed spine model can be an excellent solution.

Our study has shown that the use of the life-size 3D-printed spine model improves accuracy and length of time to complete pedicle screw instrumentation. Practicing instrumentation of pedicle screws on the 3D-printed spine model has shown a learning effect (Fig. 5). Less violation of the screws was seen as residents continued practicing the technique on the model. The mean total violation percentage for a resident decreased from 30% with the first spine model to 10% after completing five spine models (50 pedicle screws). This violation percentage became stable at 10% from the sixth to tenth models. The mean critical violation (> 2-mm breach) percentage also decreased as residents continued practicing and became stable at 0% after completing five models. The earlier and later performed 10 spine models showed less percent total violations in the latter 10 spine models (10/100 pedicle screws) compared with the former 10 spine models (27/100 pedicle screws) (*p* < 0.05; odds ratio, 0.30; 95% CI, 0.137–0.661). Moreover, the length of time required to complete screw instrumentation decreased as residents continued to practice the skills on the 3D-printed models. The mean length of time to complete 10 pedicle screw instrumentations in one spine model was 42.8 ± 5.3 min and 35.6 ± 2.9 min for the former (100 pedicle screws) and latter 10 spine models (100 pedicle screws), respectively. The latter 10 spine models required significantly less time than the former 10 models (*p* < 0.001). A strong negative relationship was observed between repetitive time of screw instrumentation and length of procedure (Spearman’s correlation coefficient, − 0.71; *p* < 0.001).

In the study, L5 did not show any screw violation compare with 32.5% of total violation in L1. Pedicles in the lower vertebral level showed less percent violation compared with the higher vertebral level (Spearman’s correlation coefficient, − 0.28; *p* < 0.01). Pedicles of the lower vertebral level are known to be wider than the higher vertebral level, and this can be the reason for the lower rate of screw violation in the lower vertebral level pedicles. Furthermore, total violation rate (18.5%) in this study on the lumbar vertebra was lower than that (29%) on the thoracic vertebra in different cadaveric studies [8]. This may have a similar reason, as pedicles in lumbar vertebra are wider than those in the thoracic vertebra. It can be inferred that narrower pedicles have a higher possibility of screw violation than wider pedicles. Therefore, pedicle instrumentation on a young patient with congenital deformity may have higher risk for screw violation. However, practicing the skills on young cadavers with spine deformity is almost impossible because most cadavers are old aged. 3D printing enables training on a life-size replica of deformed spine or young-aged spine, which is a great advantage over using cadavers for skills training.

3D printing has a number of applications in medicine, and we propose that this technique can be used for training of pedicle screw instrumentation [19, 20]. Wu et al. provided a protocol for replication of accurate 3D spine models and reported that the models can be suitable for spinal fixation research [21]. For an in-office production of 3D models, Schwartz et al. reported it took an initial investment of $52,000 to $56,000, which covers the printer, printer base cabinet, installation, training, and printer software, plus a 1-year warranty [29]. To lower the cost, open-source software for the procedures is available [30]. The cost is expected to decrease with incremental improvements in 3D printing technology, coupled with increasing competition in the market [17].

Limitation of using 3D-printed models for pedicle screw instrumentation is that the osseous feel may be different from the real pedicle. Various materials can be used for 3D printing, and further evaluation to mimic the osseous feel of the real pedicle may enable replication of the real osseous feeling replicas in the near future.

## Conclusion

A life-size 3D-printed spine model can be an excellent tool for training beginners of the free-hand pedicle screw instrumentation.

## Abbreviations

3D printing: Three-dimensional printing
CT: Computed tomography
