# Correction to: Use of a life-size three-dimensional-printed spine model for pedicle screw instrumentation training

**DOI:** 10.1186/s13018-021-02429-y

**Published:** 2021-05-08

**Authors:** Kook Jin Chung, Hyong Nyun Kim

**Affiliations:** Department of Orthopaedic Surgery, Kangnam Sacred Heart Hospital, Hallym University College of Medicine, 948-1, Dalim-1dong, Youngdeungpo-gu, Seoul, 150--950 South Korea

**Correction to: J Orthop Surg Res 13, 86 (2018)**

**https://doi.org/10.1186/s13018-018-0788-z**

Following the publication of the original article [1], the authors found out mistake on the authorgroup section. Jung Hoon Shin who is not listed in the authors' list carried out the study but passed away before the submission of the study. The authors want to thank him for his contribution to the study. Kook Jin Chung who is not listed in the authors' list contributed to the conception of the study and carried out the study and should be the corresponding author. Hyun Jin Park, Chunyu Wang, and Kyung Ho Choi did not contribute to the study. The corrected author group is shown above.
